# Supplementary material for: Draft genome assembly and transcriptome data of the icefish Chionodraco myersi reveal the key role of mitochondria for a life without hemoglobin at subzero temperatures
Source: Commun Biol. 2019 Nov 29;2:443. doi: 10.1038/s42003-019-0685-y (PMC6884616; doi:10.1038/s42003-019-0685-y)
Supplement: Supplementary file 1 — Supplementary Information [file 42003_2019_685_MOESM1_ESM.pdf]

Supplementary Figures

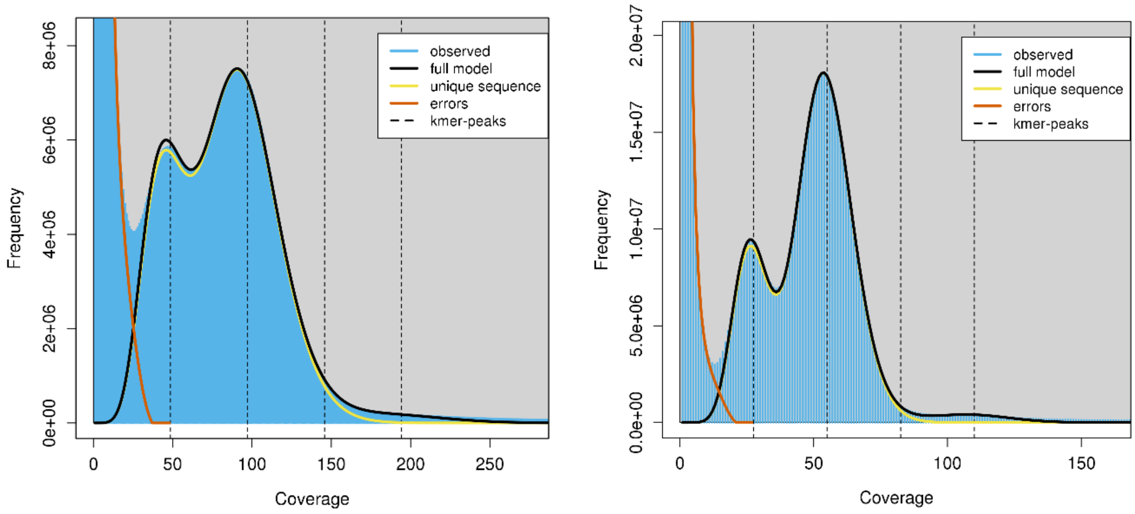

Supplementary Figure 1. K-mer (k=21) distribution histogram for *Chionodraco myersi* (left) and *Chionodraco hamatus* (right)

Supplementary Tables

Supplementary Table 1. Summary of results from BUSCO assessment for *Chionodraco myersi* and *Chionodraco hamatus*. Four other Antarctic species are also reported for comparison

	C. myersi	*C. hamatus*	*D. mawsoni*	*E. maclovinus*	*C. aceratus*	*N. coriiceps*
Number of total BUSCO groups searched*	4,584	4,584	4,584	4,584	4,584	4,584
Complete BUSCOs (%)	86.8	66.8	97.2	95.0	87.0	80.6
Complete and single copy (%)	82.1	65.4	94.8	91.6	82.7	78.6
Complete and duplicated (%)	4.7	1.4	2.4	3.4	4.3	2.0
Fragmented BUSCOs (%)	6.7	16.4	0.9	1.3	5.6	12.5
Missing (%)	6.5	16.8	1.9	3.7	7.4	6.9

*Actinopterygii dataset

Supplementary Table 2. Statistics of sequenced reads and scaffolds

	C. myersi	*C. hamatus*
Assembly method	MaSuRCA	CLC Genomics Workbench
Total base pairs	1,120,110,929	829,550,236
# of scaffolds	63,605	94,665
N50 scaff length	48,322	51,057
Max scaff length	689,883	685,670
# of Illumina raw reads	894,596,612	689,078,664
# of Illumina total base pair	135,084,088,412	82,689,439,680
# of PacBio sub reads	3,296,962	-
# of PacBio total base pairs	19,277,995,766	-

Supplementary Table 3. Genome assembly statistics (contigs and scaffolds)

	C. myersi	*C. hamatus*
Number of scaffolds	63,605	94,665
Total size of scaffolds	1,120,110,929	829,550,236
Longest scaffold	689,883	685,670
Shortest scaffold	500	500
Number of scaffolds > 1K nt	52,668 (82.8%)	55,846 (59.0%)
Number of scaffolds > 10K nt	25,498 (40.1%)	15,482 16.4%)
Number of scaffolds > 100K nt	1,916 (3.0%)	1,508 (1.6%)
Number of scaffolds > 1M nt	0 (0.0%)	0 (0.0%)
Mean scaffold size	17,610	8,763
Median scaffold size	6,752	1,253
N50 scaffold length	48,322	51,057
L50 scaffold count	5,467	3,829
scaffold %A	28.95	18.30
scaffold %C	20.87	12.69
scaffold %G	20.92	12.69
scaffold %T	28.96	18.31
scaffold %N	0.29	38.01
scaffold %non-ACGTN	0.00	0.00
Percentage of assembly in scaffolded contigs	11.8%	94.4%
Percentage of assembly in unscaffolded contigs	88.2%	5.6%
Average number of contigs per scaffold	1.1	3.7
Average length of break (>25 Ns) between contigs in scaffold	904	1,229
Number of contigs	66,856	347,646
Number of contigs in scaffolds	6,118	306,626
Number of contigs not in scaffolds	60,738	41,020
Total size of contigs	1,116,931,945	515,255,834
Longest contig	689,883	72,556
Shortest contig	151	151
Number of contigs > 1K nt	55,166 (82.5%)	147,918 (42.5%)
Number of contigs > 10K nt	26,043 (39.0%)	3,692 (1.1%)
Number of contigs > 100K nt	1807 (2.7%)	0 (0.0%)
Number of contigs > 1M nt	0 (0.0%)	0 (0.0%)
Mean contig size	16,707	1,482
Median contig size	6,492	813
N50 contig length	45,409	2,706
L50 contig count	5,826	48,551
contig % non-ACGTN	0.00	0.00
Sequencing Coverage	~100x	~80x
Sequencing Technology	Illumina + PacBio	Illumina

Supplementary Table 4. Summary of Genomescope analysis

	C. myersi		*C. hamatus*	
	Min	Max	Min	Max
Heterozygosity	1.12%	1.13%	0.77%	0.77%
Genome Haploid Length	703,517,082	704,231,362	786,726,704	786,979,902
Genome Repeat Length	199,900,085	200,103,043	258,098,672	258,181,738
Genome Unique Length	503,616,997	504,128,318	528,628,032	528,798,164
Model Fit	95.08%	98.49%	94.95%	98.55%
Read Error Rate	1.37%	1.37%	0.64%	0.64%

Supplementary Table 5. OrthoFinder analysis statistics

Number of genes	461,376
Number of genes in orthogroups	435,149
Number of unassigned genes	26,227
Percentage of genes in orthogroups	94.3
Percentage of unassigned genes	5.7
Number of orthogroups	21,718
Number of species-specific orthogroups	169
Number of genes in species-specific orthogroups	724
Percentage of genes in species-specific orthogroups	0.2
Mean orthogroup size	20.0
Median orthogroup size	19.0
G50 (assigned genes)	23
G50 (all genes)	22
O50 (assigned genes)	5,086
O50 (all genes)	5,665
Number of orthogroups with all species present	7,034
Number of single-copy orthogroups	1,169

Supplementary Table 6. RELAX analysis to identify relaxation of purifying selection on protein-coding genes. Seven strictly one-to-one orthologs encoding important erythropoietic factors were considered analysed across six teleost fish (*D. rerio, O. niloticus, G. aculeatus, E. maclovinus, D. mawsoni and C. myersi*)

Gene Symbol	OG ID	K	P-value
**GFI1B**	OG0013377	0.11	0.074
**RHAG**	OG0012154	1.48	0.311
**TAL1**	OG0008473	0.72	0.598
**LMO2**	OG0012084	0.35	0.304
**ALAD**	OG0008657	0.29	0.284
**CPOX**	OG0010190	11.60	0.853
**PPOX**	OG0004679	0.69	0.579
**Overall**	-	1.14	0.329

*K<1= relaxed selection; K>1=intensified selection.

Supplementary Table 7. Fragmentation reduction of Antarctic genome assemblies by BLASTP analysis against Stickleback proteome dataset. Only proteins covering at least 60% of the orthologous sequence in stickleback were retained in the final datasets

Species	Total genes	Matches >60% with Stickleback	Not-Matching with Stickleback	Retained genes
E. maclovinus	24,229	15,030	5,996	21,026
C. aceratus	30,773	17,046	7,854	24,900
D. mawsoni	22,516	16,122	3,463	19,585
N. coriiceps	32,331	19,786	1,826	21,612
C. myersii	38,140	20,308	6,497	26,805

Supplementary Table 8. Accession numbers of NGS data employed in the present study

Genome sequencing and assembly					
**Species**	**Reference**	**Tissue**	**N° samples**	**Accession number**	**Instrument**
Chionodraco myersi	Present study	Muscle	1	SRR8197048	PacBio RS II
Chionodraco myersi	Present study	Muscle	1	SRR8197047	Illumina HiSeq 4000
Chionodraco hamatus	Present study	Muscle	1	SRR8197057	Illumina HiSeq 4000
Genome annotation					
**Species**	**Reference**	**Tissue**	**N° samples**	**Accession number**	Illumina HiSeq 4000
Chionodraco myersi	Present study	Muscle	5	SRR8197054 to SRR8197058	Illumina HiSeq 4000
Chionodraco myersi	Present study	Spleen	1	SRR8197050	Illumina HiSeq 4000
Chionodraco myersi	Present study	Kidney	1	SRR8197052	Illumina HiSeq 4000
Chionodraco myersi	Present study	Liver	1	SRR8197049	Illumina HiSeq 4000
Chionodraco myersi	Present study	Brain	1	SRR8197051	Illumina HiSeq 4000
Comparative transcriptomic analysis					
**Species**	**Reference**	**Tissue**	**N° samples**	**Accession number**	
Chionodraco myersi	Present study	Muscle	5	SRR8197054 to SRR8197058	Illumina HiSeq 2000
Danio rerio	Hartig et al. 2016[1]	Muscle	5	SRR3381852, SRR3381854, SRR3381855, SRR3381858, SRR3381861	Illumina HiSeq 2500
Oreochromis niloticus	Unpublished	Muscle	5	ERR1940547, ERR1940637, ERR1952960, ERR1952962, ERR1953052	Illumina HiSeq 2500
Gasterosteus aculeatus	Shama et al. 2016[2]	Muscle	5	ERR1250322, ERR1250323, ERR1250324, ERR1250325, ERR1250327	Illumina NextSeq 500
Parachaenichtys charcoti	Unpublished	Muscle	2	ERR2587182, ERR2587181	Illumina HiSeq 2000
Nototenia coriiceps	Shin et al. 2014[3]	Muscle	1	SRR1015898	Illumina HiSeq 2000
Dissostichus mawsoni	Chen et al. 2019[4]	Muscle	1	SRR6794064	Illumina HiSeq 1500

Supplementary Methods

Genomic libraries preparation and sequencing

Chionodraco myersi and *Chionodraco hamatus* samples used in this study were collected in the Eastern Weddell Sea at depths between 370 and 450 m during two RV Polarstern Cruises (PS82 and PS96) coordinated by the Alfred Wegener Institute, Helmholtz Centre for Polar and Marine Research (Bremerhaven, Germany). High–molecular weight genomic DNA was extracted from both species using a Genomic-tip 100/G (Qiagen) according to manufacturer's specifications. For DNA sequencing, two genomic libraries were constructed from sheared genomic DNA (insert length 350 bp) following the standard protocol of the TruSeq DNA sample preparation kit (Illumina, CA, USA) and sequenced on an Illumina HiSeq4000 instrument following a 150 paired-end (PE) strategy. PacBio library and sequencing (8 SMRT cells) was performed for *C. myersi* by Cold Spring Harbor Laboratory (Cold Spring Harbor, NY, USA). Raw Illumina reads and PacBio subset reads were deposited in the NCBI SRA repository under the accession numbers reported in Supplementary Table 8.

RNA-seq libraries preparation and sequencing

Total RNA was extracted from spleen, kidney, liver, brain, and skeletal muscle using the RNAeasy Mini Kit (Qiagen, Hilden, Germany) according to the manufacturer's instructions. Five biological replicates were employed for muscle RNA extractions while one replicate was used for the other tissues. RNA concentration and integrity were measured with a NanoDrop ND-1000 spectrophotometer and assessed through a Bioanalyzer 2100 instrument (Agilent Technologies, Santa Clara, CA, USA). A total of nine non-normalized libraries for RNA sequencing experiments were prepared by using the SureSelect Strand Specific RNA-Seq Library Preparation kit (Agilent Technologies) and a PE sequencing was carried out on an Illumina HiSeq2500 (Illumina Inc., San Diego, CA, USA). Raw Illumina reads were deposited in the SRA repository under the accession numbers reported in Supplementary Table 8.

Reads quality analysis and filtering

For both species, quality of Illumina raw reads was analyzed with the FastQC v0.11.6 program (http://www.bioinformatics.babraham.ac.uk/projects/fastqc/). Subsequently, low-quality regions and adapters were trimmed using Trimmomatic v0.36[5]. In order to improve the quality of *C. myersi*

PacBio sequences, a hybrid error correction method was performed with the software LoRDEC v0.3 (Long Read DBG Error Correction)[6], using as reference set the Illumina short reads of the same individual.

De novo genome assembly

Chionodraco myersi

Reads obtained from Illumina and PacBio Rs II for *C. myersi* were assembled with a hybrid strategy using the MaSuRCA v3.2.8 genome assembler[7]. Scaffolds shorter than 500 bp were removed as they are of limited use and probably artifacts.

Chionodraco hamatus

Global assembly of the Illumina reads obtained for *C. hamatus* was accomplished with the software CLC Genomics Workbench v10 (https://www.qiagenbioinformatics.com/) using a minimum contig size of 500 bp and default settings for the other parameters. To remove mitochondrial contamination from the resulting assemblies a BLASTN search (e-value threshold = 1E-5) against the complete *C. myersi* and *C. hamatus* mitochondrial genomes (NCBI acc numbers: NC_029737.1 and NC_010689.1) was performed.

Genomes quality assessment

To obtain genome assembly statistics, the Assemblathon2 script was used[8] and contig break was set to 25 bp. To provide measures for quantitative assessment of the genome assembly, a Benchmarking Universal Single-Copy Orthologs (BUSCO v.3)[9] analysis was performed, based on an evolutionarily informed expectation of gene content. The Actinopterygii dataset, containing 4,584 well-conserved genes, was employed to investigate the completeness of the assembly.

To estimate the size, repeat content and heterozygosity of the genomes, a K-mer analysis (K=21) was conducted using Genomescope[10] with DNA PE libraries.

Genome masking

Repetitive elements were identified using RepeatModeler v1.0.11 (http://www.repeatmasker.org/RepeatModeler) and used to search against a fish protein database without transposon proteins to ensure exclusion of gene fragments. The fish database was downloaded from the NCBI GenBank repository selecting all the proteins belonging to the Teleostei class and filtered removing proteins with a similarity to transposon sequences (BLAST program

against RepBase database (https://www.girinst.org/repbase/) setting a e-value cutoff of 1E-5). The search was performed using the BLAST v2.8 program[11] and setting an e-value cutoff of 1E-10. Sequences with a significant match to genes were removed along with 50 bp upstream and downstream of the BLAST hit using the program ProtExcluder.pl v. 1.2 available at http://www.hrt.msu.edu/uploads/535/78637/ProtExcluder1.2.tar.gz. If the remaining sequences were shorter than 50 bp, the entire sequence was excluded.

After this filtering step, 1,420 and 705 different repetitive elements were identified in *C. hamatus* and *C. myersi*, respectively. Repetitive elements were then used to mask the species genomes using RepeatMasker v4.08 (http://www.repeatmasker.org) leading to 9.18% (C. *hamatus*) and 38.41% (*C. myersi*) of genome masked. This difference in percentage of masking is probably due to a difference in the genome assembly quality. The use of PacBio long reads in *C. myersi* assembly likely helped in resolving repetitive elements.

Gene prediction

Gene prediction was performed considering several sources of evidence: i) RNA-seq data; ii) nucleotide and protein alignments; iii) *de novo* gene training and prediction. A total of nine RNA-seq libraries coming from five tissues were used for gene prediction.

Trimmomatic[5] was used for adapter clipping. The minimum read length was set to 35 bp and a minimum quality score of 20 within a sliding window of 5 was enforced. RNA-seq reads were aligned against the reference genome using the GSNAP v2017 program[12] with default parameters and enabling the detection and alignment of spliced reads. Genome-guided transcript reconstruction was performed using StringTie v1.3.3, setting the minimum junction coverage to 3 (option –j)[13]. The transcripts were further assembled using the PASA v2.3.3 software[14], a eukaryotic genome annotation tool that exploits spliced alignments of expressed transcript sequences to automatically model gene structures. *Ab initio* gene prediction was performed using five different programs: Augustus v2.5[15,16], Snap[17], GeneID v1.4.4[18], Glimmer v3.0[19], and GeneMark v4.30[20]. GeneMark was run providing introns coordinates from RNA-Seq read alignments. GeneID was run using the *Tetraodon nigroviridis* gene model. Snap, Glimmer, and Augustus were trained using the gene models generated by PASA. Briefly, PASA alignment assemblies were used to automatically extract protein coding regions for generating a high-quality data set for training *ab initio* gene predictors. Previous collected evidence was combined for gene prediction using the EvidenceModeler v1.1.1 (EVM) program[14] in order to obtain a single gene model. The EVidenceModeler software combines

ab initio gene predictions together with protein and transcript alignments into weighted consensus gene structures. In order to reduce false positive prediction and improve the overall gene prediction quality, several filters were applied:

1) Genes predicted only by *ab initio* programs: these genes were considered good only if confirmed by at least four different *ab initio* programs, if they were complete (with a start and a stop codon) and longer than 300 base pairs.
2) Gene supported only by external evidence (*e.g.* proteins/RNA-seq): they need to be confirmed by at least two different lines of evidence or by one external evidence and at least three different *ab initio* gene predictors.
3) Predicted genes with a low *ab initio* support (filter described at step 1) were further processed. Genes supported by less than four *ab initio* programs were searched against a database of teleost protein sequences. The fish database was downloaded from the NCBI GenBank repository selecting all the proteins belonging to the Teleostei class.

   Proteins with a sequence coverage match higher than 70% and an e-value lower that 1E-20 were recovered.

Gene annotation

BLASTp[11] similarity searches (e-value threshold of 1E-5) of *C. myersi* and *C. hamatus* predicted genes were performed against the non-redundant protein database (NCBI). InterProscan5[21] was used to obtain conserved protein domains and functional annotation. The database used were PROSITE patterns, PRINTS, PFAM, PRODOM, SMART, TIGRFAM, and PANTHER. Gene Ontology and KEGG classifications were predicted running BLAST2GO 2.6.0[22] on the BLASTp and InterProscan outputs.

Supplementary Notes

C. myersi and *C. hamatus* genomes assembly and annotation

A total of 894,596,612 paired-end reads and 3,296,962 subreads, consisting of 135 and 19 Gbp, were obtained for *C. myersi* using Illumina and PacBio RS II, respectively (Supplementary Table 2).

Using MaSuRCA, these reads were assembled into 63,605 scaffolds of approximately 1.12 Gbp. The N50 and L50 statistics were 48,322 and 5,467 for the scaffolds, 45,409 and 5,826 for the broken scaffolds (i.e. contigs, see Supplementary Table 3). The quality of the genome, assessed with BUSCO (Supplementary Table 1), highlighted a percentage of 86.8% complete BUSCO groups (82.1% in single copy).

In parallel, 689,078,664 paired-end Illumina reads, for a total of 83 Gbp, were obtained for *C. hamatus.* A total of 94,665 scaffolds were assembled using the software CLC Genomics Workbench. The N50 and L50 statistics were 51,057 and 3,829 for the scaffolds, 2,706 and 48,551 for the broken scaffolds (Supplementary Table 3). The quality of the genome assessed with BUSCO (Supplementary Table 1), highlighted a percentage of 66.8% complete BUSCO groups (65.4% in single copy).

The estimated genome size by k-mer distribution for *C. myersi* and *C. hamatus* were 704 and 787 Mb respectively. See Supplementary Table 4 and Supplementary Figure 1 for detailed results of Genomescope analysis.

Genome annotation was based on similarity and experimental evidence from RNA-seq data produced in the present study (Supplementary Table 8). The total number of estimated protein-coding genes was 38,127 and 27,111 for *C. myersi* and *C. hamatus,* respectively.

A total of 24,649 of *C. myersi* protein-coding genes were assigned preliminary functions with BLASTp, and Gene Ontology (GO) terms were associated to 15,648 (63%) predicted genes based on BLASTp results and InterproScan, encompassing biological processes (2,599 (17%)), cellular components (2,585(16%)), and molecular functions (10,464 (67%)).

Genome annotation of *C. hamatus* assigned a total of 19,834 protein-coding genes to preliminary functions with BLASTp, with Gene Ontology (GO) terms assigned to 14,864 (75%) predicted genes based on BLASTp results and InterproScan, encompassing molecular functions (9,973 (67%)), biological processes (2,565 (17%)) and cellular components (2,326 (16%)).

Supplementary References

1. Hartig, E. I., Zhu, S., King, B. L. & Coffman, J. A. Cortisol-treated zebrafish embryos develop into pro-inflammatory adults with aberrant immune gene regulation. *Biol Open* **5**, 1134–1141 (2016).
2. Shama, L. N. S. *et al.* Transgenerational effects persist down the maternal line in marine sticklebacks: gene expression matches physiology in a warming ocean. *Evolutionary Applications* **9**, 1096–1111 (2016).
3. Shin, S. C. *et al.* The genome sequence of the Antarctic bullhead notothen reveals evolutionary adaptations to a cold environment. *Genome Biol.* **15**, 468 (2014).
4. Chen, L. *et al.* The genomic basis for colonizing the freezing Southern Ocean revealed by Antarctic toothfish and Patagonian robalo genomes. *Gigascience* **8**, (2019).
5. Bolger, A. M., Lohse, M. & Usadel, B. Trimmomatic: a flexible trimmer for Illumina sequence data. *Bioinformatics* **30**, 2114–2120 (2014).
6. Salmela, L. & Rivals, E. LoRDEC: accurate and efficient long read error correction. *Bioinformatics* **30**, 3506–3514 (2014).
7. Zimin, A. V. *et al.* The MaSuRCA genome assembler. *Bioinformatics* **29**, 2669–2677 (2013).
8. Bradnam, K. R. *et al.* Assemblathon 2: evaluating de novo methods of genome assembly in three vertebrate species. *Gigascience* **2**, 10 (2013).
9. Simão, F. A., Waterhouse, R. M., Ioannidis, P., Kriventseva, E. V. & Zdobnov, E. M. BUSCO: assessing genome assembly and annotation completeness with single-copy orthologs. *Bioinformatics* **31**, 3210–3212 (2015).
10. Vurture, G. W. *et al.* GenomeScope: fast reference-free genome profiling from short reads. *Bioinformatics* **33**, 2202–2204 (2017).
11. Altschul, S. F., Gish, W., Miller, W., Myers, E. W. & Lipman, D. J. Basic local alignment search tool. *Journal of Molecular Biology* **215**, 403–410 (1990).

12. Wu, T. D., Reeder, J., Lawrence, M., Becker, G. & Brauer, M. J. GMAP and GSNAP for Genomic Sequence Alignment: Enhancements to Speed, Accuracy, and Functionality. *Methods Mol. Biol.* **1418**, 283–334 (2016).
13. Pertea, M. *et al.* StringTie enables improved reconstruction of a transcriptome from RNA-seq reads. *Nat. Biotechnol.* **33**, 290–295 (2015).
14. Haas, B. J. *et al.* Automated eukaryotic gene structure annotation using EVidenceModeler and the Program to Assemble Spliced Alignments. *Genome Biol.* **9**, R7 (2008).
15. Stanke, M. & Morgenstern, B. AUGUSTUS: a web server for gene prediction in eukaryotes that allows user-defined constraints. *Nucleic Acids Res.* **33**, W465-467 (2005).
16. Stanke, M., Schöffmann, O., Morgenstern, B. & Waack, S. Gene prediction in eukaryotes with a generalized hidden Markov model that uses hints from external sources. *BMC Bioinformatics* **7**, 62 (2006).
17. Korf, I. Gene finding in novel genomes. *BMC Bioinformatics* **5**, 59 (2004).
18. Parra, G., Blanco, E. & Guigó, R. GeneID in Drosophila. *Genome Res.* **10**, 511–515 (2000).
19. Majoros, W. H., Pertea, M. & Salzberg, S. L. TigrScan and GlimmerHMM: two open source ab initio eukaryotic gene-finders. *Bioinformatics* **20**, 2878–2879 (2004).
20. Lomsadze, A., Burns, P. D. & Borodovsky, M. Integration of mapped RNA-Seq reads into automatic training of eukaryotic gene finding algorithm. *Nucleic Acids Res.* **42**, e119 (2014).
21. Jones, P. *et al.* InterProScan 5: genome-scale protein function classification. *Bioinformatics* **30**, 1236–1240 (2014).
22. Götz, S. *et al.* High-throughput functional annotation and data mining with the Blast2GO suite. *Nucleic Acids Res.* **36**, 3420–3435 (2008).
