# Supplementary material for: Draft genome assembly and transcriptome data of the icefish Chionodraco myersi reveal the key role of mitochondria for a life without hemoglobin at subzero temperatures
Source: Commun Biol. 2019 Nov 29;2:443. doi: 10.1038/s42003-019-0685-y (PMC6884616; doi:10.1038/s42003-019-0685-y)
Supplement: Supplementary file 2 — Description of Additional Supplementary Files [file 42003_2019_685_MOESM2_ESM.docx]

**SUPPLEMENTARY FILES**

**Supplementary Data 1.** **Putative duplicated OGs and the corresponding annotations in *C. myersi*, *C. aceratus, D. mawsoni* and *N. coriiceps*.**

See *Supplementary Data 1.xlsx*
