# Supplementary material for: Draft genome assembly and transcriptome data of the icefish Chionodraco myersi reveal the key role of mitochondria for a life without hemoglobin at subzero temperatures
Source: Commun Biol. 2019 Nov 29;2:443. doi: 10.1038/s42003-019-0685-y (PMC6884616; doi:10.1038/s42003-019-0685-y)
Supplement: Supplementary file 4 — Reporting Summary [file 42003_2019_685_MOESM4_ESM.pdf]

nature research

Corresponding author(s): Luca Bargelloni

Last updated by author(s): Oct 29, 2019

Reporting Summary

Nature Research wishes to improve the reproducibility of the work that we publish. This form provides structure for consistency and transparency in reporting. For further information on Nature Research policies, see Authors & Referees and the Editorial Policy Checklist.

Statistics

For all statistical analyses, confirm that the following items are present in the figure legend, table legend, main text, or Methods section.

n/a	Confirmed	
☐	☒	The exact sample size (*n*) for each experimental group/condition, given as a discrete number and unit of measurement
☐	☒	A statement on whether measurements were taken from distinct samples or whether the same sample was measured repeatedly
☐	☒	The statistical test(s) used AND whether they are one- or two-sided<br>*Only common tests should be described solely by name; describe more complex techniques in the Methods section.*
☒	☐	A description of all covariates tested
☐	☒	A description of any assumptions or corrections, such as tests of normality and adjustment for multiple comparisons
☐	☒	A full description of the statistical parameters including central tendency (e.g. means) or other basic estimates (e.g. regression coefficient) AND variation (e.g. standard deviation) or associated estimates of uncertainty (e.g. confidence intervals)
☐	☒	For null hypothesis testing, the test statistic (e.g. *F*, *t*, *r*) with confidence intervals, effect sizes, degrees of freedom and *P* value noted<br>*Give P values as exact values whenever suitable.*
☒	☐	For Bayesian analysis, information on the choice of priors and Markov chain Monte Carlo settings
☒	☐	For hierarchical and complex designs, identification of the appropriate level for tests and full reporting of outcomes
☒	☐	Estimates of effect sizes (e.g. Cohen's *d*, Pearson's *r*), indicating how they were calculated

Our web collection on statistics for biologists contains articles on many of the points above.

Software and code

Policy information about availability of computer code

Data collection	Not applicable
Data analysis	Chionodraco hamatus genome assembly was performed by using CLC Genomics Workbench 10.<br>Chionodraco myersi genome assembly was performed by using MaSuRCA genome assembler 3.2.3<br>Quantitative assessment of the genome assembly was performed with BUSCO v.3<br>Repetitive elements were identified using RepeatModeler v.1.0.8<br>Repetitive elements were identified and used to mask the genome using RepeatMasker v4.0.6<br>Gene prediction relied on several software: PASA v.2.0.2, GSNAP 2016-04-04, Augustus 3.2.2, Snap 2006-07-08, GeneId v.1.4, Glimmer v.3.0.4, GeneMark v.3.49 and EvidendeModeler v2012-06-25<br>Gene annotation was assessed by using InterProscan5 v.19.58<br>Gene Ontology and KEGG classifications were predicted running BLAST2GO 2.6.0<br>Homology relationships were reconstructed using OrthoFinder v1.1.10<br>Reads mapping for gene expression analysis was performed using STAR aligner v 2.5.3a<br>Differential gene expression was conducted using EdgeR v 3.12.1<br>Gene set enrichment analysis was carried out using GSEA 3.0

For manuscripts utilizing custom algorithms or software that are central to the research but not yet described in published literature, software must be made available to editors/reviewers. We strongly encourage code deposition in a community repository (e.g. GitHub). See the Nature Research guidelines for submitting code & software for further information.

Data

Policy information about availability of data

All manuscripts must include a data availability statement. This statement should provide the following information, where applicable:

- Accession codes, unique identifiers, or web links for publicly available datasets
- A list of figures that have associated raw data
- A description of any restrictions on data availability

Chionodraco myersi and Chionodraco hamatus genome assemblies were deposited in DDBJ/ENA/GenBank under the accession RQJG00000000 (version RQJG01000000) and RRCA00000000 (version RRCA01000000), respectively. All raw sequence data produced in this study were deposited in NCBI Short Reads Archive (SRA) under accession numbers from SRR8197047 to SRR8197058.

Field-specific reporting

Please select the one below that is the best fit for your research. If you are not sure, read the appropriate sections before making your selection.

☐ Life sciences ☐ Behavioural & social sciences ☒ Ecological, evolutionary & environmental sciences

For a reference copy of the document with all sections, see nature.com/documents/nr-reporting-summary-flat.pdf

Ecological, evolutionary & environmental sciences study design

All studies must disclose on these points even when the disclosure is negative.

Study description	The complete genome of two closely related icefish species, Chionodraco myersi and Chionodraco hamatus, was sequenced and annotated. Muscle transcriptome comparative analysis between Chionodraco miersy and other three model species (Danio rerio, Gasterosteus aculeatus, Oreochromis niloticus) were conducted on RNAseq data from five biological replicates for each species.
Research sample	Genome assembly: one muscle sample of Chionodraco myersi and one muscle sample of Chionodraco hamatus were employed for genomic libraries preparation and sequencing.<br>Genome annotation: five tissues were dissected from a Chionodraco myersi adult individual<br>Gene expression experiment: a total of five animals were used. We chose this number of samples based on literature data and on our experience in statistical analysis of RNAseq data.
Sampling strategy	No sample size calculation was necessary for genome sequencing since one sample was employed for each species. For gene expression study a total of five biological replicates were employed based on literature data.
Data collection	Genomic data were produced by Illumina and PacBio sequencing. Transcriptomic (RNAseq) data were produced by Illumina sequencing. RNA-seq data for zebrafish, tilapia, stickleback, and three species of notothenioids were downloaded from SRA GenBank.
Timing and spatial scale	Samples collection were performed from 2013 to 2015 (two RV Polarstern Cruises, PS82 and PS96). Genomic and transcriptomic data were produced in 2016. Genome assembly, annotation and statistical analyses were conducted in 2017 and 2018.
Data exclusions	No data were excluded from the analysis
Reproducibility	Biological replicates have been used and p-values obtained from statistical analyses were corrected for multiple testing (False Discovery Rate).
Randomization	This aspect is not relevant in this study since the experimental groups were based on animal species
Blinding	his aspect is not relevant since this study aimed at finding differences between species, thus group allocation could not be blinded. Notably, during data collection and analysis, all samples have been processed in the same condition.

Did the study involve field work? ☐ Yes ☒ No

Reporting for specific materials, systems and methods

We require information from authors about some types of materials, experimental systems and methods used in many studies. Here, indicate whether each material, system or method listed is relevant to your study. If you are not sure if a list item applies to your research, read the appropriate section before selecting a response.

Materials & experimental systems

n/a	Involved in the study
☒	☐ Antibodies
☒	☐ Eukaryotic cell lines
☒	☐ Palaeontology
☐	☒ Animals and other organisms
☒	☐ Human research participants
☒	☐ Clinical data

Methods

n/a	Involved in the study
☒	☐ ChIP-seq
☒	☐ Flow cytometry
☒	☐ MRI-based neuroimaging

Animals and other organisms

Policy information about studies involving animals; ARRIVE guidelines recommended for reporting animal research

Laboratory animals	The study did not involve laboratory animals
Wild animals	Chionodraco myersi and Chionodraco hamatus samples used in this study were collected in the Eastern Weddell Sea at depths between 370 and 450 m during two RV Polarstern Cruises (PS82 and PS96) coordinated by the Alfred Wegener Institute, Helmholtz Centre for Polar and Marine Research (Bremerhaven, Germany). Animals were sacrificed by anesthetic overdose soon after capture, no housing or experimental condition were applied.
Field-collected samples	Chionodraco myersi and Chionodraco hamatus samples used in this study were collected in the Eastern Weddell Sea at depths between 370 and 450 m during two RV Polarstern Cruises (PS82 and PS96) coordinated by the Alfred Wegener Institute, Helmholtz Centre for Polar and Marine Research (Bremerhaven, Germany). Animals were sacrificed by anesthetic overdose soon after capture, no housing or experimental condition were applied.
Ethics oversight	No ethical approval was required

Note that full information on the approval of the study protocol must also be provided in the manuscript.
